# 8-Prenylkaempferol Suppresses Influenza A Virus-Induced RANTES Production in A549 Cells via Blocking PI3K-Mediated Transcriptional Activation of NF-**κ**B and IRF3

**DOI:** 10.1093/ecam/nep066

**Published:** 2011-06-16

**Authors:** Wen-Fei Chiou, Chen-Chih Chen, Bai-Luh Wei

**Affiliations:** ^1^National Research Institute of Chinese Medicine, Taipei 112, Taiwan; ^2^Institute of Life Science, Collage of Science and Engineering, National Taitung University, Taitung, Taiwan; ^3^Institute of Traditional Medicine, School of Medicine, National Yang-Ming University, Taipei, Taiwan

## Abstract

8-Prenylkaempferol (8-PK) is a prenylflavonoid isolated from *Sophora flavescens*, a Chinese herb with antiviral and anti-inflammatory properties. In this study, we investigated its effect on regulated activation, normal T cell expressed and secreted (RANTES) secretion by influenza A virus (H1N1)-infected A549 alveolar epithelial cells. Cell inoculation with H1N1 evoked a significant induction in RANTES accumulation accompanied with time-related increase in nuclear translocation of nuclear factor-**κ**B (NF-**κ**B) and interferon regulatory factor 3 (IRF-3), but showed no effect on c-Jun phosphorylation. 8-PK could significantly inhibit not only RANTES production but also NF-**κ**B and IRF-3 nuclear translocation. We had proved that both NF-**κ**B and IRF-3 participated in H1N1-induced RANTES production since NF-**κ**B inhibitor pyrrolidinedithio carbamate (PDTC) and IRF-3 siRNA attenuated significantly RANTES accumulation. H1N1 inoculation also increased PI3K activity as well as Akt phosphorylation and such responsiveness were attenuated by 8-PK. In the presence of wortmannin, nuclear translocation of NF-**κ**B and IRF3 as well as RANTES production by H1N1 infection were all reversed, demonstrating that PI3K-Akt pathway is essential for NF-**κ**B- and IRF-3-mediated RANTES production in A549 cells. Furthermore, 8-PK but not wortmannin, prevented effectively H1N1-evoked I**κ**B degradation. In conclusion, 8-PK might be an anti-inflammatory agent for suppressing influenza A virus-induced RANTES production acts by blocking PI3K-mediated transcriptional activation of NF-**κ**B and IRF-3 and in part by interfering with I**κ**B degradation which subsequently decreases NF-**κ**B translocation.

## 1. Introduction

Respiratory virus infection causes airway inflammation and bronchial asthma exacerbation [1]. During influenza virus infection, monocytes/macrophages and lymphocytes were found to selectively infiltrate into the infected tissues. The mechanism has been investigated, and virus-infected airway epithelial cells, at least in part, contribute to it by expressing various chemokines that attract inflammatory cell infiltration [2]. The production of regulated on activation, normal T cell expressed and secreted (RANTES, a chemokine belong to CCL5) has been found in nasal secretions of patients suffering from upper respiratory tract infection with influenza virus and is implicated in the pathogenesis of airway during influenza virus infection [3]. Thus, drugs that prevent leukocytes invasion by interfering with RANTES secretion may serve as potential agents for interrupting the pathogenesis after viral infection [4].

The root of *Sophora flavescens* (Kushen) has been used in traditional herbal preparations in China for centuries, and is prescribed as an antiviral agent [5]. By means of the cytopathologic effect (CPE) assay, Ma et al. [6] found that the active extract of *S. flavescens* possesses antiviral property against respiratory syncytial virus (RSV). Song et al. [7] studied the antiviral effects of 120 extracts from 39 herbs using both plaque-forming assay and cytotoxicity assay and proved that *S. flavescens* possessed more powerful antiviral effects than other herbs. A variety of bioactive compounds isolated from *S. flavescens* have been reported to exhibit antiviral activity. For example, quinolizidine alkaloids isolated from this herb showed potent anti-hepatitis B virus (anti-HBV) activity against HBsAg secretion [8]. Moreover, two flavonoids of *S. flavescens* (sophoraflavanone G and Kurarinone) have been reported to have the ability of inhibiting inflammatory responsiveness such as chemotaxis and cyclooxygenase II induction [9, 10]. 8-Prenylkaempferol (8-PK) is a prenylflavonoid isolated from *S. flavescens*. Our previous study demonstrated that 8-PK can inhibit bacteria endotoxin-evoked inflammatory response in macrophages by preventing proinflammatory proteins (inducible nitric oxide synthase and cyclooxygenases II) induction (P.-C. Tsai et al., unpublished data). However, whether 8-PK blocks proinflammatory chemokine such as RANTES synthesis when confronted by viral infection remained unclear.

Multiple distinct signaling pathways are activated by virus infection, leading to phosphorylation events that induce the activation of NF-*κ*B and AP-1 and subsequent activation of specific target genes [11]. Expression of RANTES is also regulated primarily at the level of transcription, and their gene promoter regions contain recognition sites for many virus-activated transcription factors. In addition to NF-*κ*B and AP-1, the interferon regulatory factor (IRF-3) pathway controls a distinct, but interrelated, arm of the inflammatory response to viral infection [11]. Virus infection induces phosphorylation of IRF-3 on specific C-terminal serine residues and permits cytoplasmic-to-nuclear translocation of IRF-3. Lin et al. [12] reported that RANTES gene transcription can be directly induced by IRF-3 and suggested that IRF-3 immunoregulatory target genes at least contain this chemokine.

RANTES produced by virus-infected alveolar epithelial cells has been implicated in various pathophysiological processes including inflammation. Therefore, inhibition of RANTES expression by interfering with transcription factors activation had been considered as potential anti-inflammatory agents. In this study, we demonstrated that 8-PK decreases RANTES production in influenza virus (H1N1)-infected human alveolar epithelial cell by interfering with nuclear translocation of NF-*κ*B and IRF-3. We also investigated the upstream signaling mechanisms on how 8-PK regulated activation of transcription factors to inhibit RANTES production.

## 2. Methods

### 2.1. Isolation of 8-PK

The dried, chipped roots of *S. flavescens* (8 kg), collected from Hualien, Taiwan, were extracted with MeOH (80 l × 3). The combined extracts were evaporated in vacuum to give a black residue, which was suspended in water (10 l) and centrifuged (10 000 g, 30 min) to give water-soluble and water-insoluble portions. The MeOH-soluble material of water-insoluble portion was chromatographed repeatedly using silica gel, Sephadex LH-20, preparative TLC and HPLC to afford 8-PK, yellow prisms and other 28 compounds [13]. The structure of 8-PK was elucidated with mass and NMR spectra and in comparison with published data [14]. The purity of 8-PK was >98% as determined by HPLC and ^1^H-NMR.

### 2.2. Cell Culture

Madin-Darby canine kidney (MDCK, American Type Culture Collection ATCC, CCL-34, Rockville, MD, USA) cells were grown in DMEM (Gibco BRL, Grand Island, NY, USA) supplemented with 10% heat-inactivated, virus- and mycoplasma-free fetal calf serum (FCS, Biological Industries, Kibbutz Beit Haemek, Israel), 100 U/mL of penicillin, 100 *μ*g/mL of streptomycin, and 2 mM l-glutamine (Biological Industries, Israel). A549 alveolar epithelial cells (ATCC CCL-185, USA) were grown in RPMI 1640 medium (Gibco BRL) as described previously [15].

### 2.3. Virus Preparation, Infection and Viral Growth Assay

Influenza A virus strain A/PR/8/34 (H1N1) was propagated in MDCK cells in serum-free DMEM containing porcine trypsin (Sigma Chemicals Co., St Louis, MO, USA). For viral infection, confluent monolayer of A549 cells were inoculated with H1N1 at a multiplicity of infection (MOI) of one plaque forming unit (PFU)/cell in serum-free RPMI 1640 medium as described previously [15]. After a 1-h absorption period, the virus-containing medium was removed and cells were further incubated in the absence or presence of tested drugs, respectively. For RANTES assay, supernatants were collected at 48 h then measured using commercially available ELISA kits (R&D Systems, Inc., Minneapolis, MN, USA) according to the procedures provided by the manufacture. All samples were determined in triplicate. Cell viability was determined by Alamar Blue Kit Assay (Serotec Ltd., Oxford, UK) as described previously [16]. For western blot analysis, protein was extracted at 1, 6 and 16 h post-infection (p.i.). For virus growth assay, confluent monolayers of A549 cells in 24-well plates were infected with H1N1 at 0.1 MOI for 1 h then replaced with medium containing 8-PK of various concentrations. Viruses were harvested at 24, 36 and 48 h p.i using a process through three cycles of freezing and thawing, and clarified by low speed centrifugation (500 g for 10 min). Virus yields in the culture supernatants were assessed by the standard plaque assay in MDCK cells as described previously [17]. As a control, the infected cells incubated in 8-PK-free medium were included throughout the experiment.

### 2.4. Small Interfering RNA and Transfections

IRF-3 (GenBank accession no. NM-001571) was targeted using a predesigned ON-TARGET plus SMARTpool Small Interfering RNA (siRNA) (catalog no. L-006875-00-0020, Dharmacon). Nontargeting siRNA was used as a control in an identical manner as that of relevant siRNA. A549 cells were transfected at 70% confluence with a final concentration of 100 nM SMARTpool siRNA or nonspecific control pool using DharmaFECT transfection reagents (Dharmacon) according to the manufacturer's instructions. Forty-eight hours after transfection, cells were virus infected for the times indicated in the figure legends. The efficiency of siRNA silencing was evaluated using western blot analysis. In another set of experiment, culture medium was collected for RANTES measurement after siRNA transfection.

### 2.5. Preparation of Nuclear and Cytoplasmic Extracts for Western Blot Analysis

Nuclear and cytoplasmic extracts of A549 cells were prepared using NE-PER nuclear and cytoplasmic extraction reagent (Pierce, Rockford, IL, USA) according to the manufacturer's instruction supplemented with a set of protease inhibitors (Pierce). Protein concentration was determined using BCA reagents (Pierce) according to the manufacturer's instructions. Thirty micrograms of cytoplasmic protein extracts or 20 *μ*g of nuclear protein extracts were denatured in Laemmli buffer and separated using 8% SDS—polyacrylamide gel electrophoresis. After transferring, membrane was detected by antibodies against NF-*κ*B p65; I*κ*B-*α*, IRF-3 (all purchased from Santa Cruz Biotechnology, CA, USA), Akt and c-Jun (both purchased from Cell Signaling Technology, Beverly, MA, USA). Then it was incubated with secondary antibody (Amersham, Buckinghamshire, UK) and detected by ECL (Amersham). Results were expressed as fold of control. Nucleolin (Chemicon Intermational Inc.) served as a loading control for nuclear extracts, and actin (Chemicon Intermational Inc.) as a loading control for cytoplasmic extracts.

### 2.6. Immunoprecipitation of PI3K and ELISA for Detection of PI3K Activity

Protein extracts in the absence or presence of 8-PK, wortmannin or LY294002 were collected at 16 h p.i. After centrifugation at 10 000 g for 10 min at 4°C, the supernatant (cell lysates) were incubated with anti-PI3K p85 antibody (Santa Cruz Biotechnology), followed by addition of a 50% slurry of protein A-agarose beads in PBS for 1 h at 4°C. The beads were washed three times in wash buffer 1 [buffer A containing 1% (vol-/vol-) Nonidet P-40], three times with wash buffer 2 [0.1 M Tris-HCl (pH 7.4), 5 mM LiCl and 0.1 mM sodium orthovanadate] and twice in wash buffer 3 [10 mm Tris-HCl (pH 7.4), 150 mM LiCl, 5 mM EDTA and 0.1 mM sodium orthovanadate]. The immunoprecipitates were immunoblotted with anti-p85 antibodies to confirm the equal amounts of p85 protein were used in the assays. On the other hand, PI3K activity was measured *in vitro* using a competitive ELISA format (Echelon Biosciences, Inc., Salt Lake City, UT, USA) according to the manufacturer's instructions. The result was expressed as percentage of control.

### 2.7. Statistical Analysis

All values in the text and figures represent mean ± SE. The data were analyzed by one-way analysis of variance (ANOVA) followed by *post-hoc* Dunnett's *t*-test for multiple comparisons. Values of *P* < .05 were considered significant.

## 3. Results

### 3.1. 8-PK Inhibited H1N1-Induced RANTES Production

**Figure 1:**
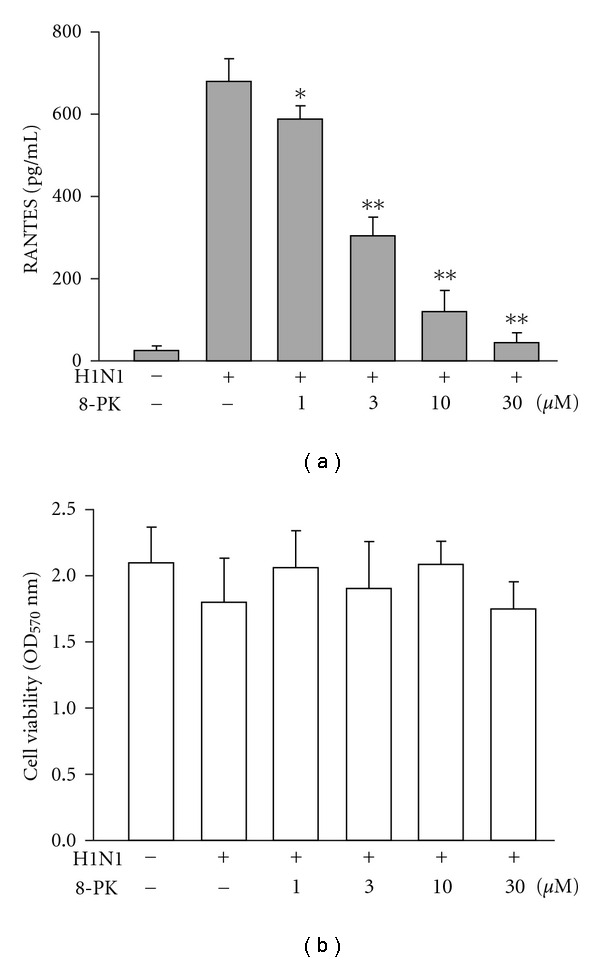
Effects of 8-PK on (a) RANTES production and (b) cell viability in H1N1-infected A549 alveolar epithelial cells. RANTES concentration was assessed as described in Section 2. Data reported are mean ± SE of six independent experiments, each performed in triplicate. *P < .05 and **P < .01, indicates significant differences as compared with H1N1 inoculation alone.

At 1 h after infection, the virus-containing medium was removed and cells were further incubated in medium containing 8-PK for 48 h. As shown in Figure 1, virus infection evoked a serious accumulation of RANTES (679 ± 56 pg/mL) as compared with that of the mock group (25 ± 11 pg/mL). 8-PK (1, 3, 10 and 30 *μ*M) treatment inhibited virus-evoked RANTES production in a concentration-dependent manner: statistical significance was observed starting at 1 *μ*M (*P* < .05) and suppression approaching basal level was achieved by 30 *μ*M 8-PK. Data of cell viability assessed by MTT assay showed that 8-PK at all concentrations did not express significant cytotoxicity.

### 3.2. H1N1 Virus-Activated Nuclear Translocation of Transcription Factors and Akt Phosphorylation in A549 Cells

**Figure 2:**
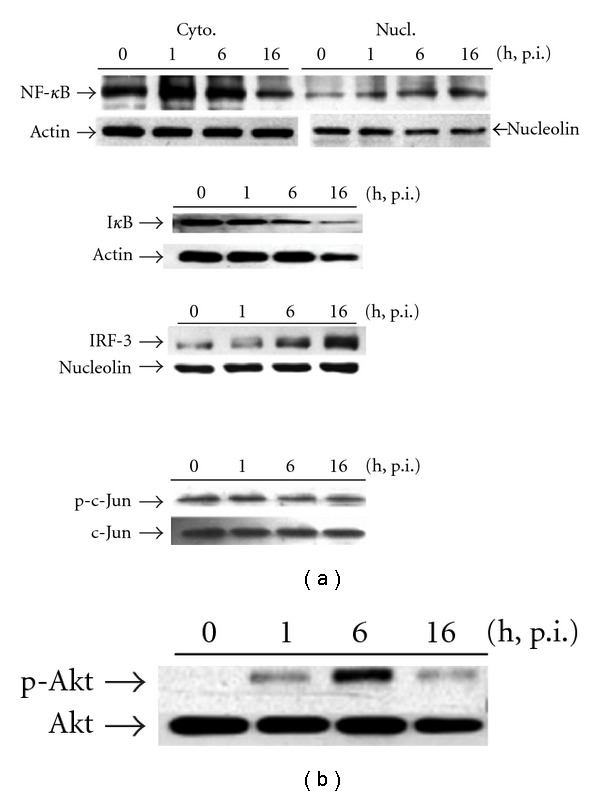
Time-related effect of H1N1 inoculation on (a) transcriptional factors activation measured by NF-*κ*B and IRF-3 nuclear translocation, I*κ*B degradation and c-Jun phosphorylation, as well as (b) Akt phosphorylation in A549 alveolar epithelial cells, respectively. Nuclear (for NF-*κ*B and IRF-3) and cytosolic proteins (for I*κ*B and c-Jun) were extracted in the absence of virus (indicated as 0) or obtained at 1, 6, or 16 h p.i as described in Section 2 then assessed by western blotting. Similar results were obtained in four independent experiments.

Cytoplasmic/nuclear protein was extracted at 1, 6 or 16 h p.i, respectively. Results depicted in Figure 2(a) (upper) indicate that nuclear NF-*κ*B (65 kD) appears already in the nucleus of the mock group, a significant nuclear translocation was increased at 1 h, reaching a maximal peak around 16 h p.i. After 24 h, a significant decrease in the level of NF-*κ*B was observed in the nucleus of infected cells perhaps due to its return into the cytoplasm (data not shown). The nuclear translocation of NF-*κ*B is regulated by the cytoplasmic inhibitor subunit I*κ*B*α* via its binding to p65. One of the major pathways for NF-*κ*B activation involves the phosphorylation of I*κ*B*α* followed by I*κ*B*α* degradation and the subsequent migration of NF-*κ*B dimers from the cytoplasm to the nucleus. Results showed an initial I*κ*B*α* (37 kD) degradation after 1 h of infection with a maximal loss at time 16 h. I*κ*B*α* slowly started to reappear into the cytoplasm at 24–36 h p.i. (data not shown).

IRF-3 is retained in the nucleus only after activation-induced phosphorylation, subsequent dimerization and translocation to the nucleus [11]. We found that IRF-3 translocated to the nucleus was enhanced in response to H1N1 infection in a time-dependent manner. As shown in Figure 2(a) (center), efficient translocation of IRF-3 to the nucleus appeared at 6 h showing peak accumulation at 16 h p.i. This was followed by a moderate loss of nuclear IRF-3 observed at 24 h p.i. (data not shown), reflecting deactivation of IRF-3 and recycling to the cytoplasm. Nucleolin served as a loading control for nuclear extract. An opposite result was observed in another transcription factor, c-Jun (a major component of activator protein-1, AP-1) [18]. No substantial increase in c-Jun phosphorylation was noticed during the 16-h p.i. period. Even after a prolonged duration of up to 48 h, no significant induction of c-Jun phosphorylation was observed.

To determine whether the phosphatidylinositol 3-kinase (PI3K)-Akt signaling cascade is also activated upon an influenza A virus infection, we investigated the specific phosphorylation of Akt at serine 473 (Ser473). Upon infection of A549 cells with H1N1, phosphorylation of Akt was observed in a biphasic manner during the 16-h p.i. period. As shown in Figure 2(b), no significant protein band appeared in lysates of mock cells; however, an obvious activation of Akt was detectable at 1 h, reached its maximum after 6 h, persisted at a somewhat lower level for up to 16 min and then declined to the basal level at 24 h p.i. (data not shown). The lower trace indicated the gel images using antibody against control Akt.

### 3.3. H1N1-Stimulated NF-*κ*B and IRF-3 Translocation as well as Akt Phosphorylation Is Associated with RANTES Production

**Figure 3:**
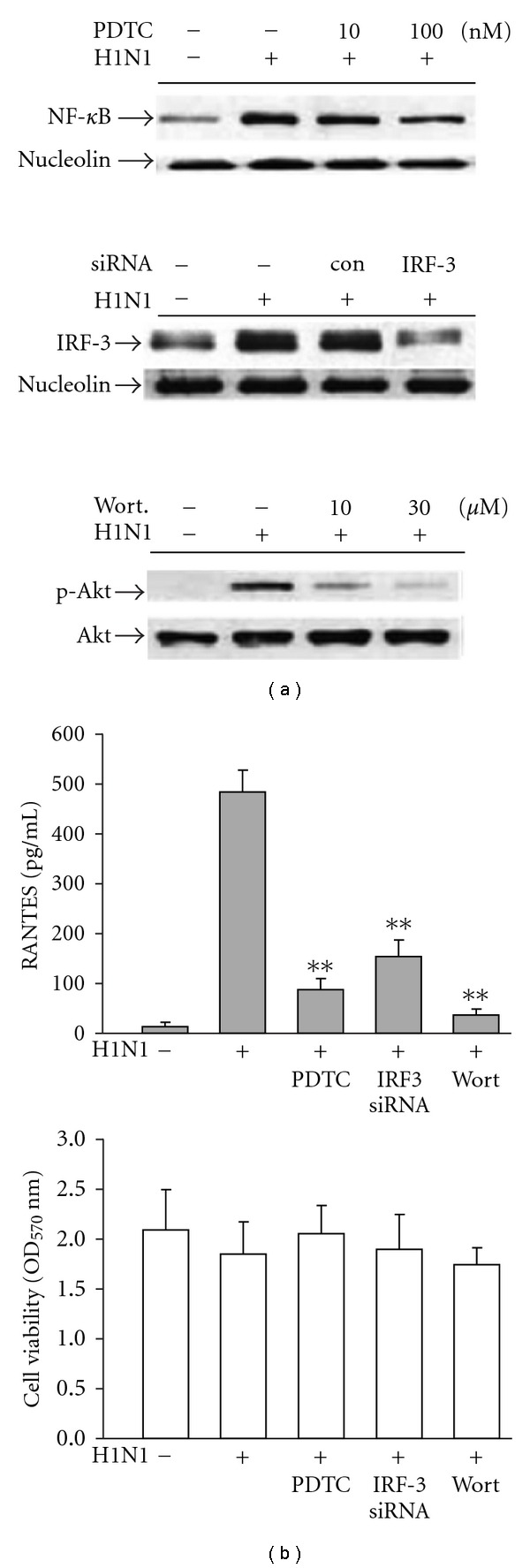
(a) Inhibitory effects of pyrrolidinedithio carbamate (PDTC, a NF-*κ*B inhibitor), IRF-3 siRNA and wortmannin (wort, a PI3K inhibitor) on the nuclear translocation of NF-*κ*B/IRF-3 and phosphorylation of Akt in H1N1-infected A549 cells and (b) the same treatment on RANTES accumulation (100 nM PDTC, 100 nM siRNA and 30 *μ*M wortmannin). For nuclear translocation assay, cells were lysed at 16 h p.i. and nuclear protein was extracted for western blot analysis as described in Section 2. While assessing Akt phosphorylation, cells were lysed at 6 h p.i. then cytosolic protein was extracted. For RANTES measurement, culture medium was collected at 48 h (p.i.). Data reported are mean ± SE of six independent experiments, each performed in triplicate. **P < .01 indicates significant differences as compared with H1N1 inoculation alone.

Although stimulating the cell with H1N1 virus resulted in nuclear translocation of NF-*κ*B and IRF-3, as well as increase in phosphorylation of Akt, whether these signal molecules indeed participated in H1N1-induced RANTES production was further studied. NF-*κ*B inhibitor ammonium pyrrolidinedithio carbamate (PDTC), siRNA targeting IRF-3 (or a nontargeting control siRNA) and PI3K inhibitor wortmannin were employed to assess, their effects on individual signal protein expression and RANTES accumulation. Results illustrated in Figure 3(a) indicated that the increased nuclear translocation of NF-*κ*B and IRF-3 as well as Akt phosphorylation evoked by H1N1 virus infection were markedly inhibited in the presence of PDTC, IRF-3-siRNA and wortmannin, respectively. The findings that virus-induced RANTES accumulation was also significantly blunted by the same treatment (Figure 3(b)) reconfirmed the roles of the abovementioned signal molecules in H1N1-induced RANTES production.

### 3.4. PI3K Mediates Transcription Factor Activation

**Figure 4:**
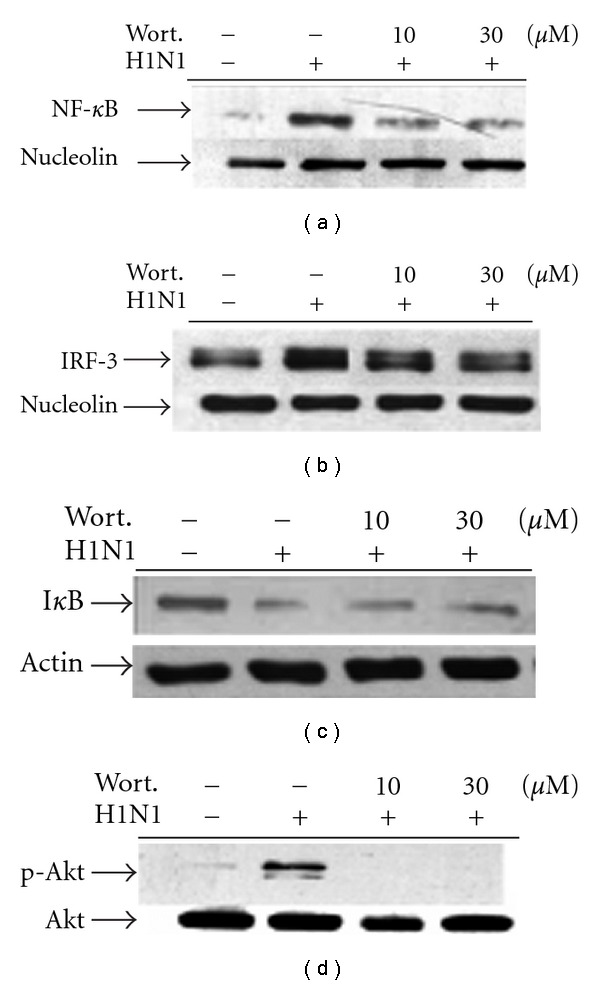
Effects of wortmannin on NF-*κ*B and IRF-3 nuclear translocation, I*κ*B degradation and Akt phosphorylation in H1N1-infected A549 cells. For nuclear translocation and cytosolic I*κ*B degradation, cells were lysed at 16 h p.i. and then protein was extracted for western blot analysis as described in Section 2. While assessing Akt phosphorylation, cells were lysed at 6 h p.i. then cytosolic protein was extracted. Similar results were obtained in three independent experiments.

To assess whether PI3K acts at upstream to regulate transcription factor activation, H1N1-induced NF-*κ*B and IRF-3 nuclear translocation in the absence or presence of wortmannin was compared. As shown in Figure 4, H1N1 inoculation strongly induced NF-*κ*B and IRF-3 nuclear translocation when observed at 16 h p.i. These phenomena were markedly attenuated in the presence of wortmannin. This provided the evidence that H1N1 virus-induced RANTES production regulated at the transcriptional level occurs strictly via the PI3K-mediated pathway. However, viral inoculation-evoked I*κ*B degradation was not affected in the presence of wortmannin.

### 3.5. Signal Pathways Involved in 8-PK-Inhibited RANTES Production

**Figure 5:**
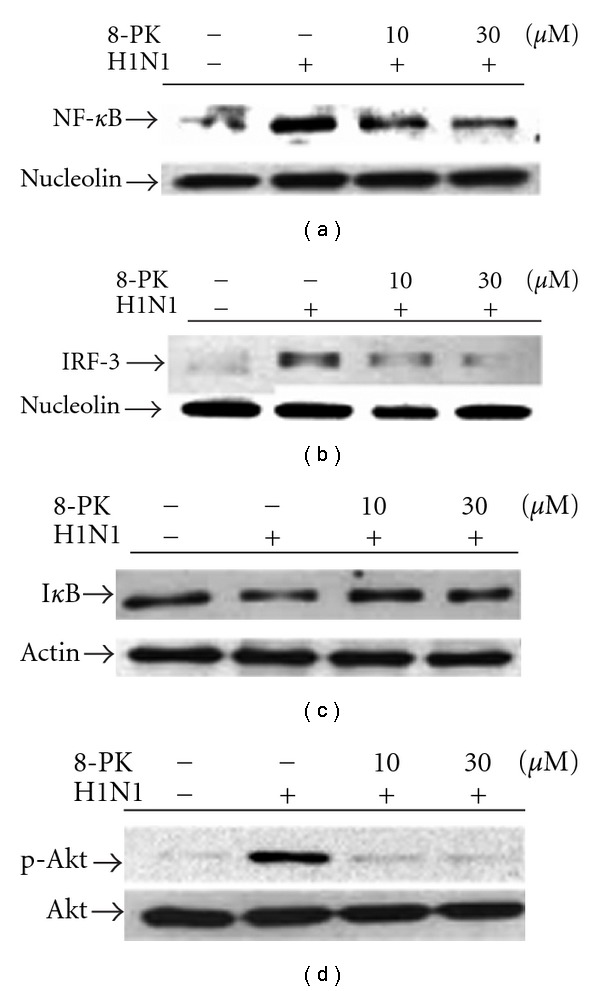
Effects of 8-PK on NF-*κ*B and IRF-3 nuclear translocation, I*κ*B degradation and Akt phosphorylation in H1N1-infected A549 cells. For nuclear translocation and cytosolic I*κ*B degradation, cells were lysed at 16 h p.i. and then protein was extracted by western blot technique as described in Section 2. While assessing for Akt phosphorylation, cells were lysed at 6 h p.i. then cytosolic protein was extracted. Similar results were obtained in four independent experiments.

Cell lysate collected in the presence or absence of 8-PK was first assessed for NF-*κ*B and IRF-3 nuclear translocation, respectively. Results shown in Figure 5 indicate that 8-PK retarded H1N1-dependent activation of both NF-*κ*B and IRF-3 translocation in a concentration-dependent manner. According to previous results, transcriptional activation of NF-*κ*B and IRF-3 is achieved through phosphorylation of upstream Akt. Thus, we tried to elucidate whether 8-PK attenuated Akt phosphorylation. Results revealed that 8-PK, as potent as wortmannin, suppressed significantly H1N1-stimulated Akt phosphorylation as potent as wortmannin (Figure 4). On the contrary, when assessed for I*κ*B*α* degradation, 8-PK but not wortmannin, had the ability to reverse H1N1-dependent degradation of I*κ*B*α*.

### 3.6. 8-PK Inhibited H1N1-Stimulated PI3K Activity

**Figure 6:**
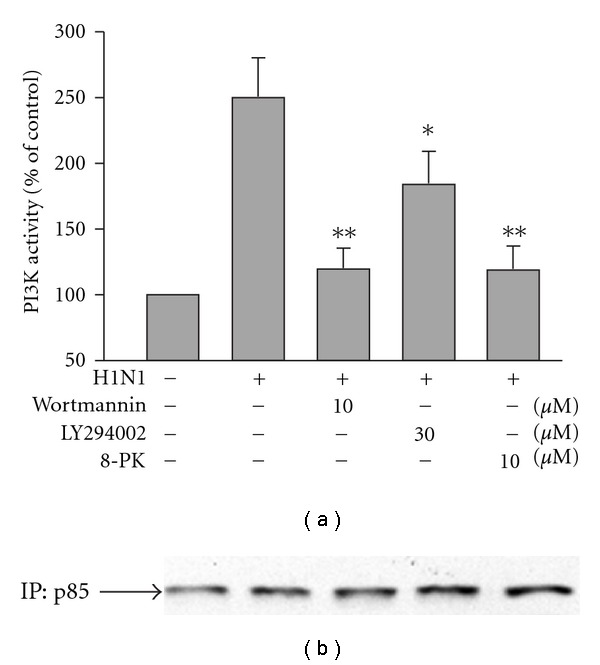
Effect of wortmannin, LY294002 or 8-PK on PI3K activity in H1N1-infected A549 cells. For PIP3 production, cells were lysed at 16 h p.i. and then protein was extracted for immunoprecipitation and ELISA assay as described in Section 2. Data reported are mean ± SE of three independent experiments, each performed in triplicate. *P < .05 and **P < .01, indicates significant differences as compared with H1N1 inoculation alone. Western blot analysis of the immunoprecipitates using anti-p85 antibodies confirmed that equal amounts of p85 protein were used in the assays (b).

To confirm the involvement of PI3K in the action of 8-PK, we further examined the effect of 8-PK on H1N1-induced PI3K activity by evaluating PIP3 production. The results showed that H1N1 inoculation increased A549 cells PI3K activity (Figure 6(a)). In fact, PIP3 production was ∼250% higher in H1N1-infected cell when compared to non-infected control cells, suggesting that H1N1 inoculation significantly increased PI3K-mediated PI3P formation in A549 cells. The increased in PIP3 production was suppressed in the presence of PI3K inhibitors wortmannin or LY294002. Furthermore, treatment with 8-PK also significantly attenuated H1N1 mediated PI3P formation. Western blot analysis of the immunoprecipitates using anti-p85 antibodies confirmed that equal amounts of p85 protein were used in the assays (Figure 6(b)).

### 3.7. 8-PK Did Not Affect Viral Growth

**Figure 7:**
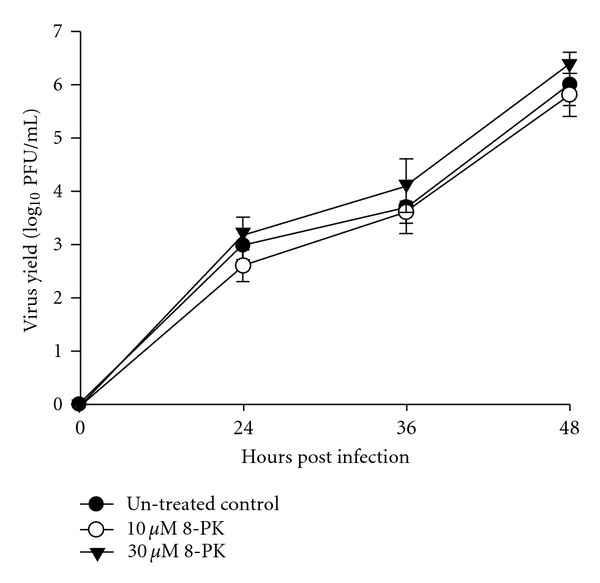
Effect of 8-PK on influenza A virus replication. A549 cells were infected with influenza A/H1N1 virus (MOI = 0.1) in the absence or presence of 8-PK. Viral yield was measured at 24, 36 and 48 h p.i. by plaque assay in MDCK cells respectively, and were represented as log_10_ PFU/mL. Data reported are mean ± SE of three independent experiments, each performed in triplicate.

To exclude the effect of 8-PK on viral replication, we analyzed the effect of 8-PK on virus yield at various times p.i. As shown in Figure 7, the change in virus yields by 8-PK was not observed at any concentration tested (10 and 30 *μ*M) at all phases of infection (24, 36 and 48 h p.i.). Resveratrol, a polyphenolic compound with the ability to inhibit influenza A virus replication [19], was employed as a positive control. We found that 30 *μ*M resveratrol reduced about 4 log units of virus yields, and this significant reduction (*P* < .001) was observed at all phases of infection (data not shown). The results suggested that 8-PK did not exert antiviral effect.

## 4. Discussion

Except tight control of viral replication/survival, it is well documented that modulation of inflammatory response through treatment with anti-inflammatory agent can effectively reduce clinical complications and to optimize recovery in virus-infected patients. Much attention has been focused on identifying the herbal remedy capable of inhibiting inflammatory process from natural resources. Proinflammatory chemokine RANTES has been reported to play a crucial role in the progression of chronic inflammation in airway after viral infection. Herein, we found that 8-PK significantly inhibited H1N1-induced RANTES accumulation in A549 cells without any obvious harmful effect on cell viability and did not directly possess antiviral activity.

Expression of most chemokines is regulated primarily at the level of transcription and is mediated by certain protein kinases, dependent on various stimuli and cell types. Virus infection leads to the activation of at least three families of transcriptional factors: NF-*κ*B, IRF-3 and AP-1. We provided evidences that IRF-3 and NF-*κ*B both played essential roles in the H1N1-induced RANTES production because cells treated with PDTC or transfected with IRF-3 siRNA all showed significantly reduced RANTES accumulation. However, the AP-1 pathway seemed to play a minor (or no) role in H1N1-induced RANTES formation because change in c-Jun phosphorylation was not observed.

The possibility of the involvement of common signaling pathways controlling IRF-3 and NF-*κ*B phosphorylation was further investigated. It has demonstrated that many viruses, including influenza A virus, activate the PI3K/Akt signal pathway [20, 21] and this pathway has been shown to be involved in CCL5 retinal expression in human pigment epithelial cells after viral infection [22]. We have herein shown that H1N1-induced RANTES formation correlated with Akt phosphorylation in A549 cells and the peak phosphorylation occurred somewhat faster than nuclear translocation of NF-*κ*B and IRF-3, suggesting that Akt signaling may occur upstream of the NF-*κ*B and/or IRF-3 pathways. In fact, inhibition of PI3K/Akt activity by wortmannin reduced significantly not only the H1N1-induced nuclear translocation of NF-*κ*B and IRF-3 but also RANTES accumulation. These data revealed that PI3K/Akt was critical in mediating transcriptional activation of NF-*κ*B and IRF-3 for RANTES synthesis in response to H1N1 infection.

It has been suggested that Akt may facilitate the phosphorylation of NF-*κ*B p65 and subsequent nuclear translocation [23, 24]. Akt phosphorylates a number of substrates at an RXRXXS/T motif, one of which is identified as IKK*α*. Li and Stark [25] have suggested that Akt activation of IKK*α* could be upstream of the phosphorylation of p65 by IKK. On the contrary, Dhawan et al. [24] reported that inhibitors of PI3K blocked the endogenous NF-*κ*B luciferase activity of malignant melanoma cells. However, these inhibitors did not block IKK phosphorylation of the I*κ*B*α* substrate, indicating that Akt-mediated NF-*κ*B activation is downstream of IKK or separated from the IKK*α*-mediated phosphorylation of I*κ*B*α*. Our result obtained from Figure 4 is consistent with the finding of Dhawan et al. that wortmannin did not affect I*κ*B*α* degradation in cells confronted by H1N1 infection.

**Figure 8:**
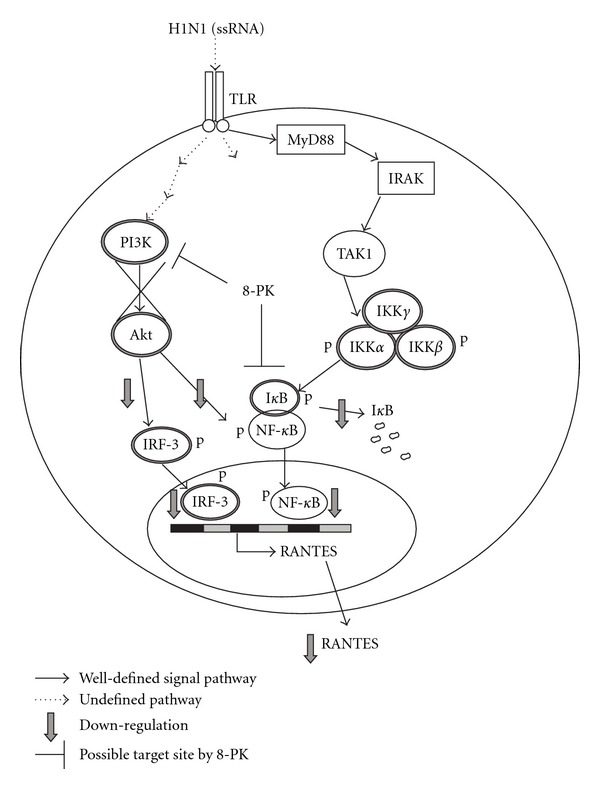
Schematic diagram illustrating the signaling pathways involved in 8-PK's inhibition of RANTES production in H1N1-infected A549 cells. After viral ssRNA binding to TLR, the adaptor protein MyD88 couples TLR and IRAK. Subsequently, MyD88 mediates a signaling cascade in TLR-triggered cells that leads to activation of IKK-I*κ*B-NF*κ*B pathways and control inflammatory genes expression include RANTES. The core IKK complex consists of the kinases IKK*α* and IKK*β* and the regulatory IKK*γ*/NEMO protein. The activation of IKK*α*/*β* depends on phosphorylation of serines at their activation loop. This process probably involves phosphorylation by recruit upstream kinases such as TAK1 leading to the rapid phosphorylation, ubiquitination, and ultimately proteolytic degradation of I*κ*B, which frees NF-*κ*B to translocate to the nucleus, where it regulates RANTES gene transcription. The suppression of H1N1-induced RANTES production by 8-PK may occur through downregulate I*κ*B degradation and NF-*κ*B nuclear translocation. Whether 8-PK interfere undefined upstream TAK1-IKK events to suppress RANTES production needed further study. An alternative pathway regulating RANTES production is via PI3K pathway. This leads to the activation of Akt, a cytosolic serine/threonine kinase that acts downstream of PI3K, and subsequently phosphorylation of IRF-3, a transcription factor that regulate RANTES gene expression. We found that 8-PK also can target at PI3K to block PI3K-mediated Akt phosphorylation and downregulate subsequently either NF-*κ*B nuclear translocation or IRF-3 phosphorylation in A549 cells reciprocally to suppress RANTES production. ssRNA, single strand RNA; TLR, Toll-like receptor; MyD88, myeloid differentiation (MyD) marker; IRAK, IL-1R-activated kinase; TRAF6, TNF*α* receptor-associated factor 6; TAK1, transforming growth factor-*β* (TGF-*β*)-activated kinase 1; RANTES, regulated on activation, normal T cell expressed and secreted.

Upon virus infection, PI3K is activated and mediates activation of the transcription factor IRF-3 [26]. Earlier findings using wortmannin indicate that inhibition of PI3K resulted in nuclear mistranslocation of IRF-3 and reduced IRF-3-dependent promoter activity as well as RANTES production. This would imply an antiviral function of the kinase in influenza virus-infected cells. Thus, PI3K is a perfect example of a seemingly antiviral signaling component. Our results showed that H1N1-evoked increase in PI3K activity was significantly attenuated by 8-PK. Thus, 8-PK may act like wortmannin to suppress PI3K activity and block subsequently the PI3K-mediated Akt phosphorylation and nuclear translocation of NF-*κ*B and IRF-3. This at least is one of the possible mechanisms for inhibiting influenza A virus-induced RANTES production by 8-PK. An arresting finding is that wortmannin did not alter I*κ*B degradation after viral infection, but 8-PK significantly prevented it. The results suggest that the inhibition of RANTES production by 8-PK could occur either by acting on the PI3K/Akt common pathway to downregulate NF-*κ*B and IRF-3 transcription or by a direct effect on I*κ*B or beyond IKK events. The proposed action mechanism by 8-PK is summarized in Figure 8. Upstream of the IKK lies multi-protein complexes that are activated by various receptor systems including virus-recognized Toll-like receptor (TLR), which sense microbial structures that are present in pathogens. In certain cases, the inflammatory process becomes dysregulated and chronic inflammatory diseases may develop. Understanding the interactions between the TLR and inflammatory signal pathways will provide further clues to the pathogeneses of these diseases and to the development of efficient therapies for combating them. More than 40 proteins participate in these complexes, depending on the receptor system activated. TLR family members signal via the adaptor proteins myeloid differentiation marker 88 (MyD88) leading to activation of the IL-1R-activated kinase (IRAK), family of protein kinases and the kinase transforming growth factor-*β* (TGF*β*)-activated kinase 1 (TAK1), which in turn, regulates IKK phosphorylation [27, 28]. Whether 8-PK also modulates these protein complexes leading to decreased inflammatory gene expression merited further study in the future.

Phytochemical studies revealed that the root of *S. flavescens* comprises mainly quinolizidine alkaloids and prenylated flavonoids [29, 30]. Previous works are mostly pharmacological and clinical research on its alkaloids. However, prenylated flavonoids from this herb also show a variety of biological and chemical activities. For example, a prenylated flavonol sophoflavescenol is a potent and selective inhibitor of cGMP phosphodiesterase 5 [31]. Regarding the tested compound 8-PK, which exerted estrogenic activity by binding to rat uterine and was an effective inhibitor of *α*-glucosidase, a key enzyme involved in diabetes type 2 [32, 33]. Our preliminary data demonstrated that 8-PK also has the ability to suppress bacteria endotoxin-evoked inflammatory response in macrophages by reducing pro-inflammatory protein (inducible nitric oxide synthase and cyclooxygenases II) expression. In the present study, we further reported that 8-PK could inhibit influenza A virus-induced RANTES production in alveolar epithelial cells which had no concern with antiviral property. Being one of the major components of Kushen, 8-PK may serve as a broad anti-inflammatory agent to prevent either bacteria- or virus-evoked pathogenesis. However, other properties of 8-PK from biological, cellular, and/or *in vivo* perspectives are worth further exploration.
